# The Impact of Biopsy on Human Embryo Developmental Potential during Preimplantation Genetic Diagnosis

**DOI:** 10.1155/2016/7193075

**Published:** 2016-01-28

**Authors:** Danilo Cimadomo, Antonio Capalbo, Filippo Maria Ubaldi, Catello Scarica, Antonio Palagiano, Rita Canipari, Laura Rienzi

**Affiliations:** ^1^GENERA Centre for Reproductive Medicine, Clinica Valle Giulia, Via G. de Notaris 2/b, 00197 Rome, Italy; ^2^Dipartimento di Scienze Anatomiche, University of Rome “La Sapienza”, Istologiche, Medico Legali e dell'Apparato Locomotore, Sezione Istologia ed Embriologia Medica, Via Antonio Scarpa 16, 00161 Rome, Italy; ^3^GENETYX, Molecular Biology Laboratory, Via Fermi 1, 36063 Marostica, Italy; ^4^Seconda Università di Napoli, Via Antonio Vivaldi 43, 81100 Caserta, Italy

## Abstract

Preimplantation Genetic Diagnosis and Screening (PGD/PGS) for monogenic diseases and/or numerical/structural chromosomal abnormalities is a tool for embryo testing aimed at identifying nonaffected and/or euploid embryos in a cohort produced during an IVF cycle. A critical aspect of this technology is the potential detrimental effect that the biopsy itself can have upon the embryo. Different embryo biopsy strategies have been proposed. Cleavage stage blastomere biopsy still represents the most commonly used method in Europe nowadays, although this approach has been shown to have a negative impact on embryo viability and implantation potential. Polar body biopsy has been proposed as an alternative to embryo biopsy especially for aneuploidy testing. However, to date no sufficiently powered study has clarified the impact of this procedure on embryo reproductive competence. Blastocyst stage biopsy represents nowadays the safest approach not to impact embryo implantation potential. For this reason, as well as for the evidences of a higher consistency of the molecular analysis when performed on trophectoderm cells, blastocyst biopsy implementation is gradually increasing worldwide. The aim of this review is to present the evidences published to date on the impact of the biopsy at different stages of preimplantation development upon human embryos reproductive potential.

## 1. Introduction

Preimplantation Genetic Diagnosis and Screening (PGD/PGS) is a tool whose application in Assisted Reproduction Techniques (ART) has significantly grown in the last decades [1]. The final aim of PGD/PGS is to define whether an embryo is affected by a monogenic disease and/or chromosomal impairments, thus preventing the implantation of a symptomatic fetus and/or limiting the risks underlying the transfer of chromosomally abnormal embryos (mainly implantation failures and miscarriages). In synthesis, PGD/PGS is a powerful tool to reach the goal of a pregnancy and attenuate its adverse events. In order to achieve this goal, it is mandatory not to significantly harm the embryo during the biopsy and to preserve its viability and reproductive potential. First do not harm is a dogma in clinical practice that perfectly applies also to this context. This review will be an overview of the different stages of embryo preimplantation development the biopsy procedure can be performed at. The definition of the technical drawbacks that could resolve in a negative influence on reproductive potential will be provided. Mainly three different approaches will be examined, namely, polar body (PB) biopsy from the mature oocyte and/or the zygote, blastomere biopsy at the cleavage stage, and trophectoderm (TE) biopsy at the blastocyst stage. Recently, morula stage biopsy has also been proposed and will be briefly discussed.

The ESHRE PGD consortium data referring to years 2009-2010 [2] highlighted an uneven distribution in the number of biopsy procedures performed in Europe between the three main strategies. In particular, blastomere biopsy alone accounted for almost 90% of the total, while TE biopsy accounted for less than 1%. The same data referring to years 2012-2013 [3] instead showed an impressive countertendency since the rate of PB biopsy and cleavage stage biopsy procedures decreased to approximately 2% and 75%, respectively. Blastocyst stage biopsy implementation instead is gradually increasing in ART. The reason for this dramatic change is a review and meta-analysis published by Mastenbroek and colleagues in 2011 clearly showing that PGS as it was conducted, namely, cleavage stage biopsy analyzed by 9-chromosome FISH, is a harmful procedure [4]. However, while 9-chromosome FISH use has been strongly reduced in favor of the more accurate and reliable Comprehensive Chromosome Screening (CCS) techniques, blastomere biopsy is still the method mainly adopted for PGD. The use of PB biopsy did not spread due to its intrinsic logistic, clinical, and technical drawbacks that compromise its accuracy, especially when compared to TE biopsy strategy.

## 2. Cleavage Stage Biopsy

Cleavage stage biopsy is normally performed on day 3 embryos with at least 6 blastomeres. The zona pellucida is opened and Ca^++^/Mg^++^-free medium is used in order to loosen cell-cell adhesion and facilitate selected blastomere removal. A less traumatic impact on the growing embryo should theoretically not affect blastulation [5]. However, this is a controversial issue since several studies in mouse showed that Ca^++^ depletion caused remodeling of the cellular cytoskeleton, inevitably impacting compaction [6–9].

The biopsy is mainly conducted following 3 methods of zona breaching, namely, laser-assisted [10], mechanical [11], and Tyrode's drilling [12]. Use of the laser-assisted method represents 75% of all biopsy procedures declared in the ESHRE PGD consortium data collection XII [2]; thus it is the mostly used approach. However, apparently all the three methods do not impact clinical outcomes, as randomized controlled trials (RCTs) on sibling embryos have shown [13–16]. Probably then the reason for the prevalence of laser-assisted method resides in the standardization and reproducibility of the hole produced within the zona pellucida, which is less operator-dependent than the use of acidified Tyrode [14]. Furthermore, besides being more precise and less time-consuming, laser-assisted hatching requires also a shorter training period. In fact, the hole created by acidified Tyrode depends on variables such as the amount of solution deposited, the time of exposure, and the operator's skills; on the contrary few preset firings are sufficient according to laser-assisted method.

It has been estimated that the temperature in the media surrounding the embryo increases to 60–80°C depending on the intensity of the laser beam [17]. However, Taylor and colleagues [18] highlighted that, even when varying the laser pulse to be between 20 and 400 mW, biopsy operators did not cause any negative effect on both technical and clinical outcomes after biopsy.

Nevertheless, zona pellucida breaching itself can impact subsequent processes along preimplantation development up to the blastocyst stage. In particular, several studies highlighted the impairment of the blastocyst hatching process, whose seriousness depends on number and size of holes produced [19–21]. The severity of this issue is exacerbated by the concurrent removal of one blastomere [22, 23]. In particular, Kirkegaard and colleagues [23] compared the development to blastocyst stage of biopsied cleavage stage embryos versus control nonbiopsied embryos through time-lapse microscopy (TLM). They highlighted that the blastocysts obtained from biopsied embryos showed delayed compaction process and hatched in a nonphysiological fashion bypassing the prolonged period of zona pellucida thinning. This translated to smaller blastocysts with thicker zona pellucida.

Blastomere biopsy is also affected by problems associated with single cell analysis, both technical (e.g., high rate of amplification failure) [24, 25] and biological. In particular, chromosomal mosaicism, namely, the presence of cells with different karyotypes within the same embryo, seems to reach its highest level at this stage of preimplantation development [26–29]. In order to compensate for this, a two-blastomere biopsy strategy has been proposed. However, this strategy could involve a depletion of the embryonic mass of about 25% and in turn impact clinical outcomes [30, 31]. On the contrary, initial evidences did not demonstrate a detrimental impact of blastomere(s) loss (after biopsy and/or cryopreservation) on deriving embryo development and implantation potential [32–35]. In fact, ESHRE guidelines in 2010 suggested that this procedure could be safely applied when embryos are composed of ≥6 cells with less than 30% of fragmentation [36].

In 2013, strong evidence against this approach arose from the paired RCT by Scott Jr. and colleagues [37] and completely changed the previous scenario. In particular, here the two best quality embryos produced within a single cohort during an IVF cycle were selected for transfer either at the cleavage or at the blastocyst stage. One embryo was randomized for biopsy without aneuploidy testing and the other was instead used as paired control. Both embryos were then transferred. In case a single embryo implanted, the biopsy fragment was submitted to aSNP-based fingerprinting, as also either fetal DNA from maternal blood or buccal DNA from the newborn after delivery. Matching results indicated that the implanted embryo was the biopsied one, whereas nonmatching that the control embryo was the one that led to the delivery. A dramatic 39% relative reduction in implantation rate was reported when cleavage stage biopsy was conducted with respect to control.

An interesting theory is that embryos at this stage of preimplantation development are relatively fragile since Embryonic Genome Activation and cell differentiation processes have not occurred yet. Thus, downstream developmental processes can be irreparably compromised by removing a cell from the embryo. Such an impact in fact reflects also in a lower blastocyst rate after cleavage stage biopsy with respect to undisturbed embryos, as reported in several papers [38–40].

Given the number of studies showing the ineffectiveness and potential impairment of cleavage stage biopsy, it is not surprising that Mastenbroek and colleagues [4] in their review and meta-analysis highlighted the failure of PGS when conducted by 9-chromosome FISH on biopsied blastomere(s).

However, many embryologists and molecular biologists remained confident about the potential of aneuploidy testing in IVF. Thus, novel strategies to achieve this objective are being pursued. At present, RCTs to assess the clinical value of blastomere biopsy when associated with CCS rather than FISH are in the pipeline (NCT01571076, NCT01950104 registered in https://www.clinicaltrials.gov/). Moreover, novel biopsy strategies are being investigated by moving either backwards or onwards along the preimplantation development timeline.

## 3. Polar Body Biopsy

Polar body (PB) biopsy on MII oocytes and/or zygotes was encouraged as a valuable alternative to blastomere biopsy [41, 42]. In some countries this was mainly due to legal reasons, since embryo biopsy is not allowed. PB biopsy is potentially less invasive than any other stage of preimplantation development, since it entails the removal of waste products of meiosis. However, the applicability of this strategy has always been under debate, as mirrored by the ESHRE PGD Consortium data. In fact, PB biopsy has been used in only 10–15% of all the procedures performed in Europe in the last decade. Nowadays this rate is further decreasing probably due to the number of studies that highlighted technical, economical, biological, and clinical deficiencies underlying the approach. For instance, Capalbo and colleagues [43] reported high false positive and negative error rates when adopting this biopsy strategy. In this regard, mitotic and paternally derived aneuploidies cannot in fact be detected. The authors underlined also that both PBs from all the MII oocytes and/or zygotes are needed regardless of their developmental potential, thus making the procedure time-consuming. This in turn increases the lab workload, another important drawback related to this strategy.

The methods for zona drilling are the same as previously described for blastomere biopsy. Several papers in the literature described their application for PBs biopsy and reported no negative effects upon quality parameters, such as oocytes lysis and activation rates, development after calcium ionophore treatment, embryo chromosome breakage incidence after Tarkowski preparation, and/or embryo development [44–47]. Neonatal outcomes after PB biopsy-based PGD are also comparable to those obtained with cleavage stage-based approach [13]. On the contrary, Levin and colleagues [48] reported a higher fragmentation rate, a lower embryo quality, a higher cleavage arrest rate, and a lower mean number of blastomeres in day 3 when PB biopsy is performed with respect to control. However, the authors did not evaluate embryo implantation potential after this biopsy approach, a limitation shared by all the papers dealing with this topic. No sufficiently powered well-controlled studies have been published that report a lack of PB biopsy impact upon embryo implantation potential. In light of this absence, the safety of the procedure still remains an arguable assumption [49]. To this regard, there are two ongoing studies currently registered on the website https://www.clinicaltrials.gov/ that deal with PB biopsy: the ESHRE ESTEEM RCT (NCT01532284) and a study by the Weill Medical College (Cornell University) (NCT01574404). Hopefully, they will report powerful data about the clinical efficiency of PB biopsy to resolve the remaining issues.

**Figure 1:**
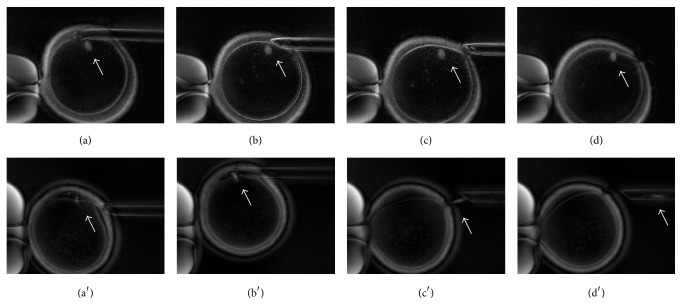
Oocyte first polar body biopsy prior to fertilization displayed through polarized light microscopy. Polarized light microscopy allows identification of the chromosome meiotic spindle (indicated by the white arrows in all figures). (a)–(d) Metaphase II oocyte first polar body biopsy with no damage to the meiotic spindle; (a′)–(d′) Telophase I oocyte first polar body biopsy with enucleation of the oocyte whose meiotic spindle remains attached to the polar body during aspiration.

A last operative concern regards whether to follow a sequential or simultaneous biopsy approach. Specifically, according to the former the PBs are retrieved at different times, while according to the latter both are retrieved at once. Even though guidelines regarding the proper timing for biopsy have not been established, it should occur between 8 and 14 hours after fertilization. By performing the biopsy before this time range there is a risk of enucleation due to spindle remnants in the second PB (Figure 1), while PB disintegration or degeneration might occur if the biopsy is performed later [42, 50]. Following a simultaneous biopsy, rather than a sequential one, a double exposure to suboptimal environmental conditions can also be prevented. However, no clinical data have been produced up to date to solve this issue.

To conclude, the application of PB biopsy has been gradually reduced in favor of TE biopsy due to the absence of reliable supporting data and the possibility of diagnostic inaccuracy.

## 4. Morula Stage Biopsy

Recently morula stage biopsy has been proposed [51]. Few data have been produced to evaluate its actual feasibility; however it is technically similar to cleavage stage biopsy and thus it shares its same drawbacks (e.g., the need for Ca^++^/Mg^++^-free buffer to loosen compaction). The main advantage of morula stage biopsy is instead shared with TE biopsy approach, namely, the number of cells retrieved. The analysis of more than a single cell leads in fact to a more robust downstream molecular investigation, which sets among the reasons that prompted blastocyst stage biopsy strategy.

## 5. Blastocyst Stage Biopsy

Blastocyst stage biopsy strategy was an important breakthrough in modern IVF. It was reported for the first time by de Boer and colleagues in 2004 [52] and the first live births following this approach were reported in 2005 by Kokkali and colleagues [53] and by McArthur and colleagues [30], respectively. Several preclinical and clinical studies soon recognized its value, so that at present it is gradually replacing both cleavage stage and PB biopsy approaches.

The power of TE biopsy resides in its higher technical and biological robustness. This approach in fact entails both lower influence of procedural errors and lower impact of mosaicism on the molecular analysis.

However, high standards are required for blastocyst culture and cryopreservation, which is an important limiting factor for the widespread implementation of this strategy. Nevertheless, once a proper culture system is set, blastocyst culture itself elicits higher live birth rate per embryo transfer than cleavage stage. This aspect in particular was highlighted by Glujovsky and colleagues in their Cochrane review of 12 RCTs [54]. Moreover, it is important to underline that also cleavage stage biopsy subtended culture to the blastocyst stage if aiming at performing a fresh embryo transfer of euploid embryos [37–39]. Thus, if any risk derived from the culture system, it would be shared by both biopsy approaches.

In order not to lose precious euploid blastocysts after warming, also an excellent vitrification program is required. In this regard, several papers in literature reported no blastocyst degeneration after biopsy [30, 55, 56] and a survival rate after warming always higher than 95% [30, 57, 58].

Following a cycle segmentation and SET policy, euploid cryopreserved blastocyst transfer also prevents hyperstimulation syndrome and multiple pregnancy [59, 60], a further important advantage.

We have recently evaluated our clinical practice across a 4-year period during which blastocyst stage PGD/PGS plus euploid SET policy was gradually implemented especially for advanced maternal age patients. Such a clinical setting did not affect the global efficacy of our treatments; namely, the pregnancy rate per oocyte retrieval was kept constant with respect to the past but increased their global efficiency. In particular, a significant increase in the overall pregnancy rate per transfer and the reduction in the multiple pregnancy rate after the implementation of this novel setting were reported [59]. In this scenario, Forman et al. [61] also demonstrated that single euploid blastocyst transfer equals the implantation rate of double untested blastocyst transfer, but it elicits better obstetrical and perinatal outcomes [62].

Finally, the paired RCT by Scott Jr. and colleagues [37] is again the real milestone that identified blastocyst stage biopsy as a procedure that does not affect embryo viability and implantation potential. TE biopsy was reported to have no impact, converse to what was found for blastomere biopsy. A possible explanation for this difference is that a smaller proportion of the whole cellular constitution of the embryo is removed, from a nonembryonic portion of the blastocyst and at a stage of preimplantation development perhaps more tolerant to manipulation.

This evidence was pivotal for the growing implementation of the novel TE biopsy CCS-based PGS policy worldwide. Dahdouh and colleagues recently underlined in a meta-analysis and in a review the positive clinical predictive value underlying this policy [63, 64]. Several RCTs are also in the pipeline and they will provide additional evidences during the next years (NCT01219283, NCT02032264, NCT02268786, NCT01977144, and NCT01917240 registered in https://www.clinicaltrials.gov/; ISRCTN81216689 registered in http://www.isrctn.com/). In particular, blastocyst stage biopsy will be evaluated together with next-generation sequencing or quantitative polymerase chain reaction analyses. The additional predictive value of the blastocyst culture system adopted and of euploid blastocyst morphology will be also investigated.

The systematic review by Lee and colleagues [65] summarized all the observational, prospective analyses and RCTs published to date. However, here the authors underlined an important limitation of many studies specifically comparing the clinical outcomes between TE and blastomere biopsy approaches. In particular, even if a higher ongoing/clinical pregnancy rate was always reported through the former approach, no correction for patient and/or embryo characteristics was provided and no prospective randomization was applied. The prospective double-blinded nonselection study by Scott Jr. and colleagues [66] is then, at least to our knowledge, the only paper properly designed to conduct this investigation. Here, by comparing the positive clinical predictive values (rate of embryos actually leading to an ongoing pregnancy after a CCS-based euploid diagnosis) of the two approaches, they confirm the higher reliability of blastocyst stage analysis with respect to cleavage stage one (48.2% versus 29.2%, *p* = 0.0016).

**Figure 2:**
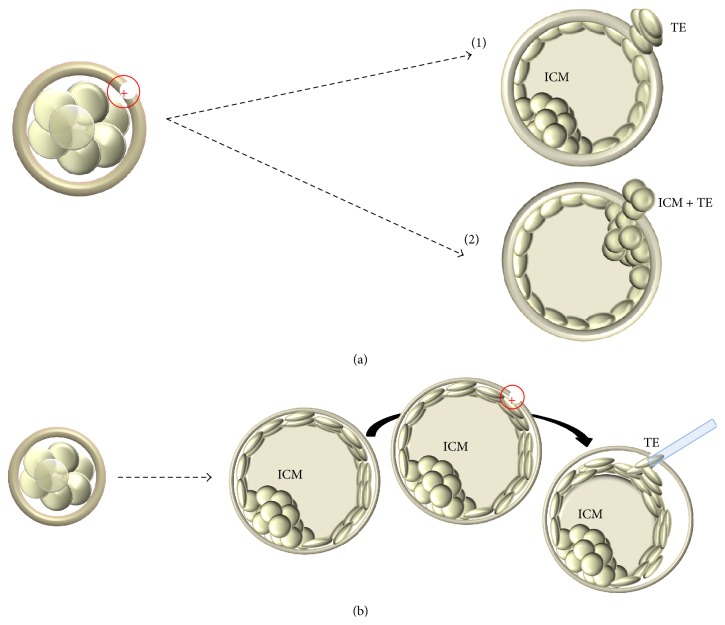
Schematic comparison between two different blastocyst biopsy approaches. (a) Day 3 hatching-based blastocyst biopsy entailing the production of a hole in the zona pellucida at the cleavage stage and the biopsy of hatching trophectoderm cells from that hole. Several pitfalls can derive a thicker zona pellucida and a smaller blastocyst since being composed by fewer and bigger trophectoderm cells. Hatching can either occur far from the ICM (a1) or involve the ICM itself impairing the procedure (a2); (b) zona opening with simultaneous blastocyst biopsy approach leaves the embryo undisturbed throughout its* in vitro* development up to the fully expanded blastocyst stage. Simultaneously opening the zona and retrieving the fragment allow the operator to choose the area and the amount of cells to biopsy. Circle with inner cross indicates laser pulse. TE, trophectoderm; ICM, Inner Cell Mass.

Nonetheless, the most commonly used strategy to conduct PGD/PGS in Europe still entails cleavage stage rather than TE biopsy. The perception of the former as less operator-dependent and more reproducible possibly underlies this tendency. In particular, embryos reach cleavage stage synchronously and are similar in terms of morphological quality and a single biopsy protocol has been described in literature. Blastocyst stage biopsy instead is characterized by a heterogeneous cohort of embryos in terms of both morphology and developmental rate, and two different methods to perform it have been published up to date. The first method has been described by McArthur and colleagues [30] and entails a hole in the zona pellucida performed in day 3 of preimplantation development. A consequent nonphysiological hatching at the blastocyst stage makes the procedure easier, but it also exposes the embryo to a potential stress along preimplantation development [23]. The second method instead was described by Capalbo and colleagues [67] and entails simultaneous zona opening and TE biopsy. The embryo is thus left undisturbed up to day 5, day 6, or even day 7, and it is biopsied exclusively after reaching full expansion (Figure 2). We investigated the accuracy and the reproducibility of this second approach associated with qPCR-based CCS. No significant differences across 7 different operators from 3 IVF centers in terms of both technical and clinical results were reported. In particular, amplification rate, qPCR data concurrence, and estimated number of cells retrieved, as well as ongoing implantation, biochemical, and miscarriage rates, were comparable [68].

Amplification rate in particular is an important parameter since a second biopsy would be needed in case of a nonconclusive result. Importantly, all the papers where TE-based CCS analysis was adopted reported always less than 3.0% of undiagnosed blastocysts [56, 58, 62, 67, 68]. This point represents a further advantage of this approach with respect to the previous single cell-based ones.

## 6. Future Perspectives

An ideal outcome would be to bypass the embryo biopsy step and predict euploidy and/or reproductive competence through noninvasive methods. For instance, static morphological evaluation, TLM criteria, and genetic/proteomic/metabolomic screening of spent IVF culture media were all proposed as promising tools. Unfortunately, several papers defined conventional parameters of embryo evaluation as just mildly correlated with aneuploidy rate [67, 69, 70], TLM as a limited tool to predict euploidy/implantation [71–73], and spent IVF culture media analysis as a fascinating theory that did not provide clinical benefit to date [74, 75].

The current perception is that noninvasive techniques could provide novel parameters to enhance our predictive power upon implantation beyond the level guaranteed by PGD/PGS, rather than trying to replace this tool.

## 7. Conclusion

Amniocentesis and chorionic villus sampling are methods available to detect vital chromosomal syndromes in the fetus during gestation; however these approaches expose women to a high risk of miscarriage ranging between 1 and 3% [76–80]. Chan and colleagues [81] underlined the importance of safety for patients by reporting that pregnant Chinese women are more prone to accept a moderate risk of undiagnosed aneuploidies than a procedure-related miscarriage risk ≥1%. Combined with the possibility of significantly reducing implantation failure and/or miscarriage rates through PGS, it is mandatory to adopt a safe biopsy method. Similarly, this need applies to PGD for monogenic mutations, a technique which requires further costs due to molecular probes construction for embryo diagnosis.

The evidence produced in the last decades extensively highlights the drawbacks of the cleavage stage approach in PGD/PGS. A significant decrease in clinical outcomes derives from the use of such a harmful biopsy strategy.

Sufficiently powered studies highlighting a similar negative impact for PB biopsy are still missing. However, this strategy suffers from important diagnostic issues leading to high false positive and false negative error rates [43].

**Figure 3:**
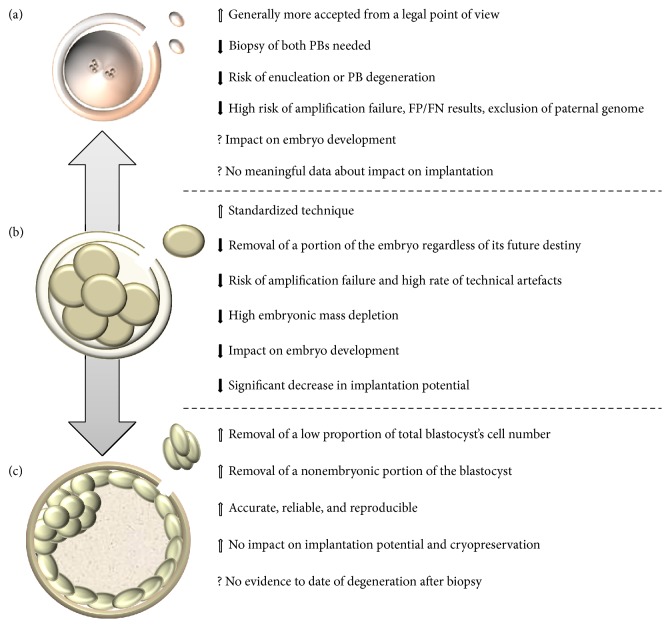
Comparison between different biopsy stages. Despite the fact that trophectoderm-based blastocyst biopsy approach (c) is not such a widespread method as cleavage stage one (b), several preclinical and clinical evidences recognized its value and highlighted its advantages with respect to the latter, as also in comparison with polar body approach (a). Black arrows indicate negative evidences described in literature; white arrows indicate positive evidences described in literature; question marks indicate still controversial aspects. PB, polar body; TE, trophectoderm; FP, false positive; FN, false negative.

The blastocyst approach instead ensures accuracy, reliability, and reproducibility and importantly shows no impact upon embryo viability and reproductive potential. The comparison of TE biopsy strategy to previous ones (summarized in Figure 3) strongly suggests that it is the most promising approach in PGD/PGS.
